# Hypotony Following Intravitreal Silicone Oil Removal in a Patient With a Complex Retinal Detachment With Giant Retinal Tear

**DOI:** 10.7759/cureus.16387

**Published:** 2021-07-14

**Authors:** Ilias Gkizis, Christina Garnavou-Xirou, Georgios Bontzos, Georgios Smoustopoulos, Tina Xirou

**Affiliations:** 1 Ophthalmology, Korgialenio-Benakio General Hospital, Athens, GRC

**Keywords:** hypotony, silicone oil removal, retinal detachment, vitrectomy, giant retinal tear

## Abstract

Postoperative ocular hypotony after silicone oil removal in complex cases of retinal detachment is a complication that can occur in about 20% of cases and can prevent the successful management of retinal detachments. Thus, it is critical to understand the mechanisms of hypotony and the potential interventions that can be done in order to avoid irreversible tissue damage. We present a case of a 35-year-old man who underwent intraocular surgery for removal of silicone oil tamponade following a combined scleral buckling and pars plana vitrectomy (PPV) surgery for a rhegmatogenous retinal detachment associated with a giant retinal tear. On Day 1 after the operation, the patient was found to have hypotony with optic disc edema, chorioretinal folds, and visual acuity of ‘hand movement’ perception. Two weeks postop, the patient’s condition stabilized, with a visual acuity of 0.38 logMAR, an intraocular pressure (IOP) of 12 mmHg, and the absence of macular edema.

## Introduction

Ocular hypotony is defined as intraocular pressure (IOP) of 5 mmHg or less. Depending on the duration and time of onset, it can be classified as acute, chronic, transient, or permanent. Whilst acute hypotony is not an uncommon phenomenon following intraocular surgery, it is generally reversible after 10-15 days [1]. Persistent cases, however, are associated with structural changes that may permanently affect vision and even lead to phthisis bulbi [2-3].

IOP regulation following vitreoretinal surgery requires constant monitoring, especially in complex cases [2]. The regulation of aqueous production and drainage might be impaired, and despite that topical medications can control IOP spikes, regular follow-up is advised to identify cases where topical therapy is insufficient [4-6]. The use of scleral buckling and silicone oil in conjunction with pars plana vitrectomy poses therapeutic challenges in the management of IOP [5]. In addition, the use of silicone oil may result in cases of postoperative IOP fluctuations. Risk factors for uncontrolled IOP include preoperative hypotony, high axial length, long-standing proliferative vitreoretinopathy (PVR), and previous intraocular surgeries, including cataract surgery [7-9].

It is essential, therefore, to understand the pathological mechanisms behind hypotony in order to prevent detrimental damage to ocular tissues. In this case, we present a patient with an acute drop of IOP following the removal of silicone endotamponade.

## Case presentation

A 35-year-old male underwent surgical removal of silicone oil tamponade in his right (OD) eye, following vitrectomy surgery for a rhegmatogenous retinal detachment with giant retinal tear (GRT). The patient’s past ocular history included high myopia (axial length=29.14 mm), and previous laser in-situ keratomileusis (LASIK) refractive surgery (10 years previously).

Sixteen months prior to the removal of the silicon oil, the patient presented to the ophthalmology department complaining of sudden painless loss of vision in his right eye. Best-corrected visual acuity (BCVA) was reduced to ‘counting fingers’ and fundoscopy had revealed a subtotal rhegmatogenous retinal detachment with a giant retinal tear (GRT) that extended ~180° temporally, from 6½ hours up to 12½ hours, with rolled posterior edges and a vitreous hemorrhage. His IOP was 12 mmHg (Goldmann applanation tonometry). At this time, the patient underwent 20G pars plana vitrectomy (PPV), with intraoperative perfluorocarbon liquid (PFCL), a 360° laser retinopexy, cryopexy to the tear’s edges, and endotamponade with silicone oil 5000-cs. The procedure had been combined with adjunctive circumferential scleral buckling.

Sixteen months after the operation, the patient’s OD BCVA was 0.12 logMAR (6/7.5 Snellen), with an IOP of 10 mmHg. The retina had remained attached. At this point, it was deemed appropriate to proceed with the removal of the silicone oil. A second 20G pars plana 3-port procedure was therefore performed, with the aid of a silicone oil removal kit (Alcon Constellation, Fort Worth, Texas). In addition, 7-0 Vicryl sutures were placed to secure the sclerotomies following removal of the oil.

On the first postoperative day, the patient was found to have substantial hypotony (IOP < 4mmHg), along with corneal edema, optic disc edema, and broad chorioretinal folds (mostly in the region of the nasal retina). BCVA was reduced to hand motion perception. The scleral wound remained sealed. At this time, it was difficult to obtain good quality fundus photographs, due to the amount of corneal edema.

**Figure 1:**
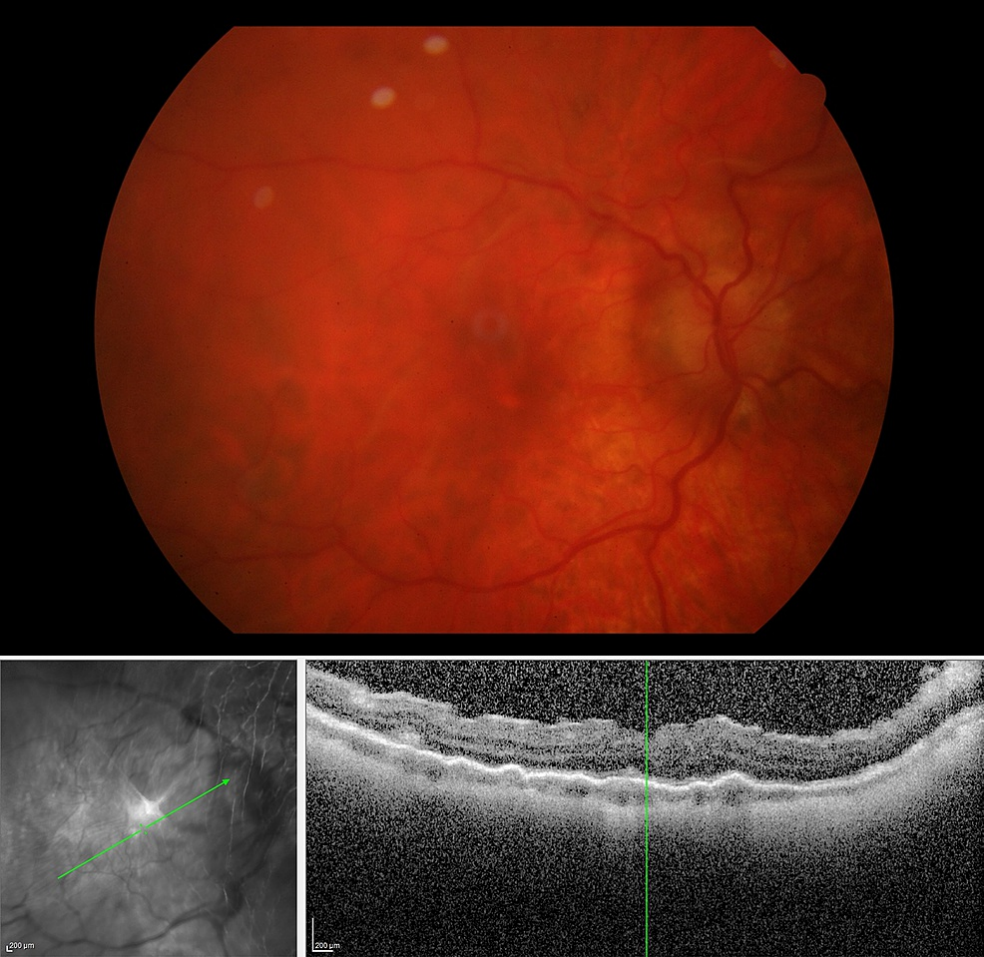
Fundus photo and OCT scan four days after silicone oil removal Upper panel: Fundus photo four days after silicone oil removal. Optic disc edema and chorioretinal folds can be observed. Lower panels: IR + OCT scans showing chorioretinal folds. OCT: optical coherence tomography; IR: infrared

**Figure 2:**
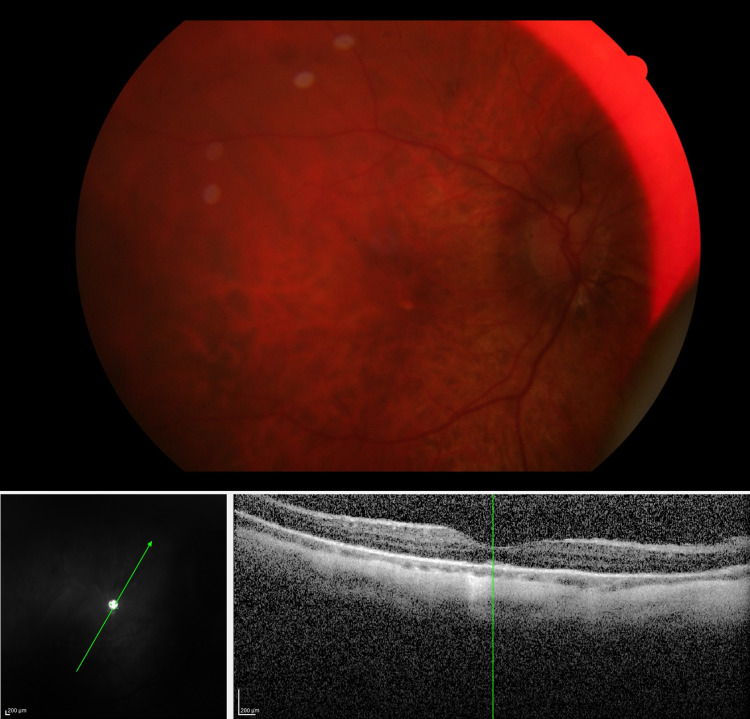
Fundus photo and OCT scan 10 days after silicone oil removal Upper panel: Fundus photo 10 days after removal of silicone oil showing resolution of optic disc edema and of chorioretinal folds. Lower panels: IR + OCT scans showing the disappearance of chorioretinal folds. OCT: optical coherence tomography; IR: infrared

During the postoperative period, the patient received a standard regimen of antibiotics and steroid drops four times a day, tapering off over a period of four weeks. He demonstrated gradual improvement (Figure 1), and two weeks after the operation, his condition had stabilized, with a BCVA of 0.38 logMAR (6/15 Snellen), IOP of 12 mmHg OD, and absence of macular edema or choroidal folds (Figure 2).

Two months later, the patient’s BCVA was 0.26 logMAR and his IOP was 14 mmHg. The fundus appearance was stable, with an attached retina and an intact macula.

## Discussion

Over the last 20 years, the field of vitreoretinal surgery has demonstrated significant improvement regarding the management of complex cases. While the incidence of PVR in GRT-related retinal detachments is higher, it is also associated with the worst surgical outcomes, the advent of perfluorocarbon liquids, wide-field viewing systems, and high-speed cutters have improved surgical outcomes. Current figures indicate a 86% success rate after a single operation and 95% success in cases where additional procedures are required [10-12]. Moreover, silicone oil is typically considered the tamponade agent of choice in complex GRT and PVR-related cases despite the potential risks of uncontrolled IOP and band keratopathy [13].

The combination of scleral buckling with pars plana vitrectomy is currently a matter of debate for complicated cases with a view to achieving optimal results while minimizing complications [14-16]. However, this approach can be performed to provide additional support to the vitreous base and to neutralize traction forces - especially in cases with PVR.

Mechanisms responsible for IOP fluctuations following vitreoretinal surgery can be divided into the following 5 categories [1]:

1. An external fistula connecting the aqueous compartment (of the anterior segment), to either the ocular surface or the subconjunctival space (as occurs in a traumatic corneal laceration, or a non-healing corneal wound).

2. An internal fistula connecting the aqueous/ or vitreous compartment to the suprachoroidal space in the presence of a corneoscleral surface. Such fistulas result from a traumatic or surgical cyclodialysis between the posterior chamber and the supraciliary space or following pars plana cyclophotocoagulation. Retinal holes and large retinal defects, such as GRTs, allow the flow of aqueous and liquified vitreous across the retinal pigment epithelium or choroid to the suprachoroidal space via an epithelial pump mechanism or due to an osmotic gradient of the aqueous humor.

3. Ciliary body insufficiency with decreased aqueous production can happen after cyclodialysis, ciliary body detachment, and/or the administration of antifibrotic agents. In complex cases of RD with anterior proliferative vitreoretinopathy (PVR), the ciliary body becomes inflamed and, often, detached with consequent hypotony.

4. Inflammation resulting from surgical or non-surgical trauma or associated with proliferative vitreoretinopathy. The mechanism of hypotony, in this case, is postulated to be a prostaglandin-mediated decrease in aqueous production (aqueous shutdown) combined with an increased uveoscleral outflow.

5. Miscellaneous non-surgical causes such as medications, ocular ischemia, etc.

Notably, circumferential buckling has also been associated with uncontrolled IOP and angle-closure [17].

Nevertheless, the patient in the case underwent an uncomplicated procedure of silicone oil removal. Hypotony in such cases is typically found in eyes already hypotonous during silicone tamponade [7,18]. It is hypothesized that silicone oil tamponade, although not affecting intraocular pressure directly, helps to maintain ocular shape. Moreover, the presence of silicone oil may impair the circulation of aqueous to the trabecular meshwork, resulting in uncontrolled IOP following its removal [18].

Acute hypotony can lead to structural remodeling of all ocular tissues [1]. The cornea becomes oedematous, with the development of characteristic folds in Bowman’s layer and Descemet’s membrane. Depending on factors like scleral rigidity, extraocular muscle insertion, and the condition of the vitreous body, the presence of such folds can lead to the striate opacity of the cornea.

The sclera, in turn, collapses and becomes deformed by the pull of the extraocular muscles, resulting in the appearance of secondary choroidal folds (the oblique muscles causing horizontal folding of the choroid and rectus muscles leading to the ‘squaring’ of the eyeball).

The iris and ciliary body become congested and ciliochoroidal detachment is often present. In mild hypotony, a ciliochoroidal detachment is limited to the anterior part of the equator. In moderate hypotony, the insertions of the vortex veins give a quadrilobed pattern to the detachment posterior to the equator. In severe hypotony, the choroidal detachment appears to be 'kissing' due to the firm posterior attachments of the short posterior ciliary vessels.

Furthermore, changes can be seen at the level of the choroid, retina, and optic nerve and can be attributed to edema, congestion, displacement, and separation of these layers. With the term chorioretinal folds, we refer to fundus wrinkling associated with parallel pale streaks. They include the inner choroid, Bruch's membrane, and the retinal pigment epithelium (RPE) and secondarily affect the overlying neurosensory retina. It is hypothesized that they are formed due to the strong connection between the choriocapillaris and the Bruch's membrane. The choroidal edema can result in folds of the overlaying Bruch’s membrane. Another suggestion is that these folds are the result of a stress-and-strain relationship between the sclera and choroid. A circumferential force following scleral thickening results in choroidal stress and the development of choroidal folds. However, the exact pathogenesis remains unclear [19].

Previous research has shown that preoperative IOP, along with ocular axial length, constitutes major risk factors for postsurgical hypotony [9]. Myopic eyes are associated with a thinned sclera, which is prone to mechanical stress during surgery and may not be able to provide adequate support for the ciliary body during surgical manipulations and intraoperative IOP fluctuations. Moreover, the ciliary body seems to be more susceptible to damage in comparison to the rest of the uvea since there is no firm attachment between the longitudinal and radial ciliary fibers and the scleral spur [20].

## Conclusions

Further research into the pathophysiology of uncontrolled IOP is required. Long-standing hypotony may result in permanent ocular damage and phthisis bulbi. Understanding the underlying mechanisms of hypotony can help clinicians manage some of the complications arising from similar vitreoretinal cases.

## References

[1] Wang Q, Thau A, Levin AV, Lee D (2019). Ocular hypotony: a comprehensive review. Surv Ophthalmol.

[2] Costa VP, Arcieri ES (2007). Hypotony maculopathy. Acta Ophthalmol Scand.

[3] Schubert HD (1996). Postsurgical hypotony: relationship to fistulization, inflammation, chorioretinal lesions, and the vitreous. Surv Ophthalmol.

[4] Anderson NG, Fineman MS, Brown GC (2006). Incidence of intraocular pressure spike and other adverse events after vitreoretinal surgery. Ophthalmology.

[5] Costarides AP, Alabata P, Bergstrom C (2004). Elevated intraocular pressure following vitreoretinal surgery. Ophthalmol Clin North Am.

[6] Tranos P, Asaria R, Aylward W, Sullivan P, Franks W (2004). Long term outcome of secondary glaucoma following vitreoretinal surgery. Br J Ophthalmol.

[7] Jonas JB, Knorr HL, Rank RM, Budde WM (2001). Intraocular pressure and silicone oil endotamponade. J Glaucoma.

[8] Henderer JD, Budenz DL, Flynn HW Jr, Schiffman JC, Feuer WJ, Murray TG (1999). Elevated intraocular pressure and hypotony following silicone oil retinal tamponade for complex retinal detachment. Incidence and risk factors. Arch Ophthalmol.

[9] Kim SW, Oh J, Yang KS, Kim MJ, Rhim JW, Huh K (2010). Risk factors for the development of transient hypotony after silicone oil removal. Retina.

[10] Berrocal MH, Chenworth ML, Acaba LA (2017). Management of giant retinal tear detachments. J Ophthalmic Vis Res.

[11] Ting DS, Foo VH, Tan TE (2020). 25-years trends and risk factors related to surgical outcomes of giant retinal tear-rhegmatogenous retinal detachments. Sci Rep.

[12] Scott IU, Murray TG, Flynn Jr HW, Feuer WJ, Schiffman JC (2002). Outcomes and complications associated with giant retinal tear management using perfluoro-n-octane. Ophthalmology.

[13] Mathis A, Pagot V, Gazagne C, Malecaze F (1992). Giant retinal tears. Surgical techniques and results using perfluorodecalin and silicone oil tamponade. Retina.

[14] Alexander P, Ang A, Poulson A, Snead MP (2008). Scleral buckling combined with vitrectomy for the management of rhegmatogenous retinal detachment associated with inferior retinal breaks. Eye (Lond).

[15] Kinori M, Moisseiev E, Shoshany N (2011). Comparison of pars plana vitrectomy with and without scleral buckle for the repair of primary rhegmatogenous retinal detachment. Am J Ophthalmol.

[16] Goezinne F, La Heij EC, Berendschot TT, Gast ST, Liem AT, Lundqvist IL, Hendrikse F (2008). Low redetachment rate due to encircling scleral buckle in giant retinal tears treated with vitrectomy and silicone oil. Retina.

[17] Chen PP, Thompson JT (1997). Risk factors for elevated intraocular pressure after the use of intraocular gases in vitreoretinal surgery. Ophthalmic Surg Lasers Imaging Retina.

[18] Zilis JD, McCuen II BW, de Juan Jr E, Stefansson E, Machemer R (1989). Results of silicone oil removal in advanced proliferative vitreoretinopathy. Am J Ophthalmol.

[19] Xirou T, Kabanarou SA, Gkizis I, Garnavou-Xirou C, Velissaris S, Theodossiadis P, Chatziralli I (2017). Chronic central serous chorioretinopathy-like maculopathy as atypical presentation of chorioretinal folds. Case Rep Ophthalmol.

[20] Hawkins WR, Schepens CL (1966). Choroidal detachment and retinal surgery. A clinical and experimental study. Am J Ophthalmol.

